# Supplementary material for: Robust models of disease heterogeneity and control, with application to the SARS-CoV-2 epidemic
Source: PLOS Glob Public Health. 2022 May 9;2(5):e0000412. doi: 10.1371/journal.pgph.0000412 (PMC10021456; doi:10.1371/journal.pgph.0000412)
Supplement: S1 File — (PDF) [file pgph.0000412.s001.pdf]

Robust models of disease heterogeneity and control, with application to the SARS-CoV-2 epidemic: supporting information

Kory D. Johnson[1◑*], Annemarie Grass[2◑], Daniel Toneian[2], Mathias Beiglböck[2], Jitka Polechová[2]

1 Institute of Statistics and Mathematical Methods in Economics, TU Wien, Vienna, Austria

2 Department of Mathematics, University of Vienna, Vienna, Austria

◑These authors contributed equally to this work.

* kory.johnson@tuwien.ac.at

Supporting Information

S1 Supplemental Figures

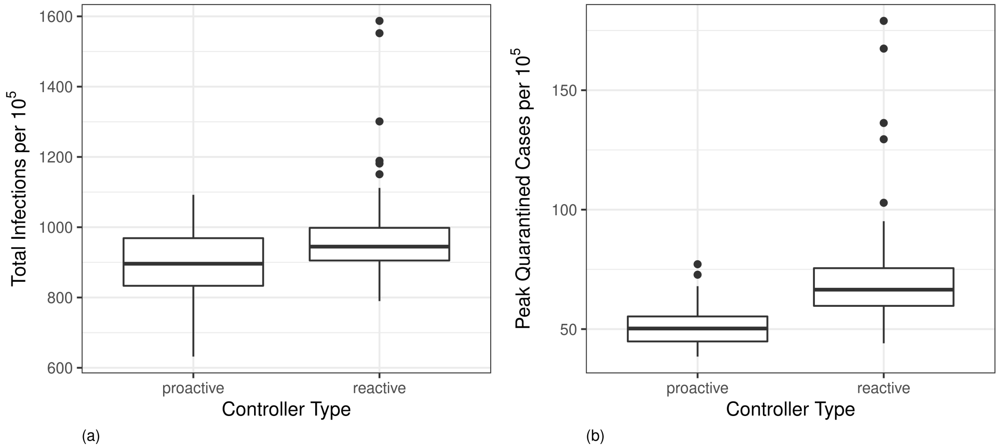

Fig A. Proactive control reduces total infections as well as peak numbers in quarantine. Simulation with Delta variant and strict case thresholds at 25-50/100,000/14 days.

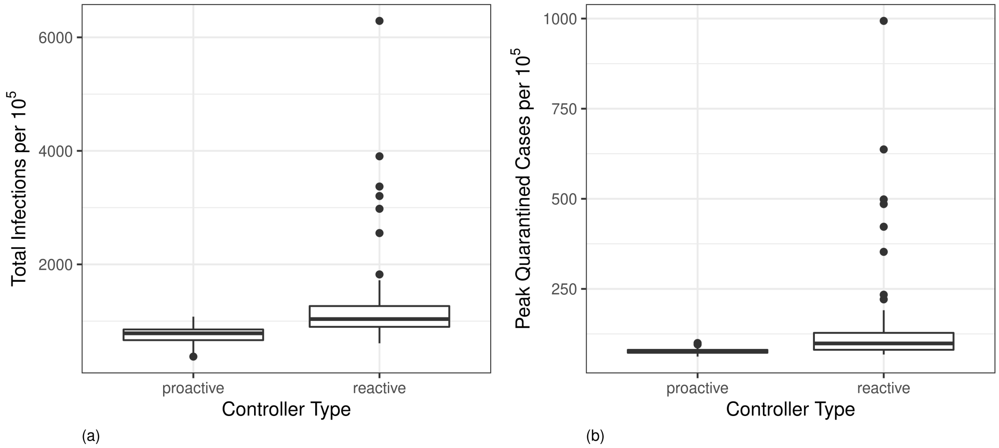

Fig B. Proactive control provides better control of statistics in all simulation runs. Simulation with Omega variant and strict case thresholds at 25-50/100,000/14 days.

We modified Omega to have higher base reproduction number as well as a shorter generation time in order to explore what more contagious variants could look like. This setting includes both waning and boosting. The reproduction number matches that of

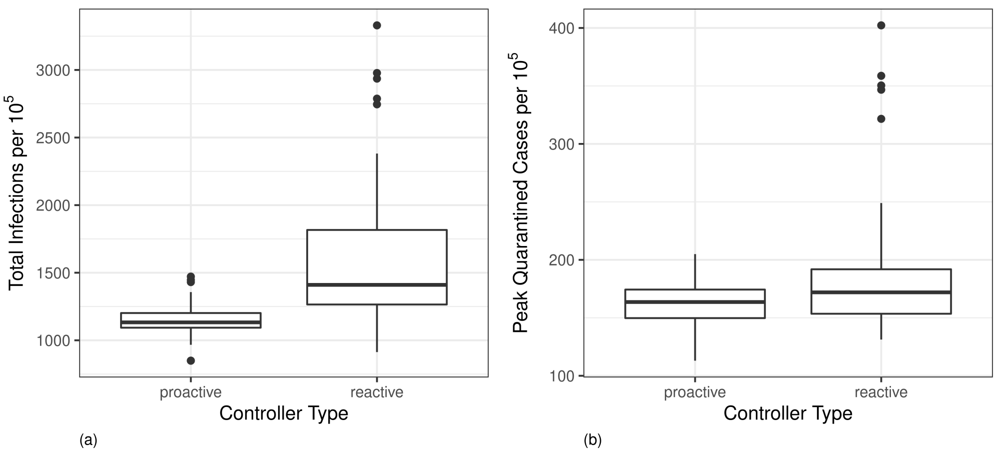

Fig C. Proactive control does not suffer due to delayed data although reactive does. Simulation with delayed controller, Omega variant, and strict case thresholds at 25-50/100,000/14 days.

Delta, with 2 and interval (1.76, 2.17). The mean of the generation interval has been reduced from original value of 4.6 to 3, with interval (1.5, 4.5). Fig F shows that winter outbreaks are observed with both a reactive and proactive controller. While the median values look quite similar, there is a significant difference in their prediction intervals.

S2 Sampling in Practice

We use the following data and parameters specific to Austria

- As generation interval (w_t) we will choose a discretization of a Gamma-distribution with mean 4.46 and standard deviation 2.63 cut off after day 13 as found by AGES in [1]. We use this generation interval for *all* variants. source:
- The Austrian population is given by $N = 8.932.664$ (as of 2021-01-01) [2].
- We use the data on the progress of the Austrian vaccination program provided by the Austrian Ministry of Health [3]. Individuals vaccinated with an mRNA respective vector vaccine will be added to the appropriate group 14 respective 21 days after the first dose. We take the median doses of the last 7 days of available data for projecting daily administered doses into the future.

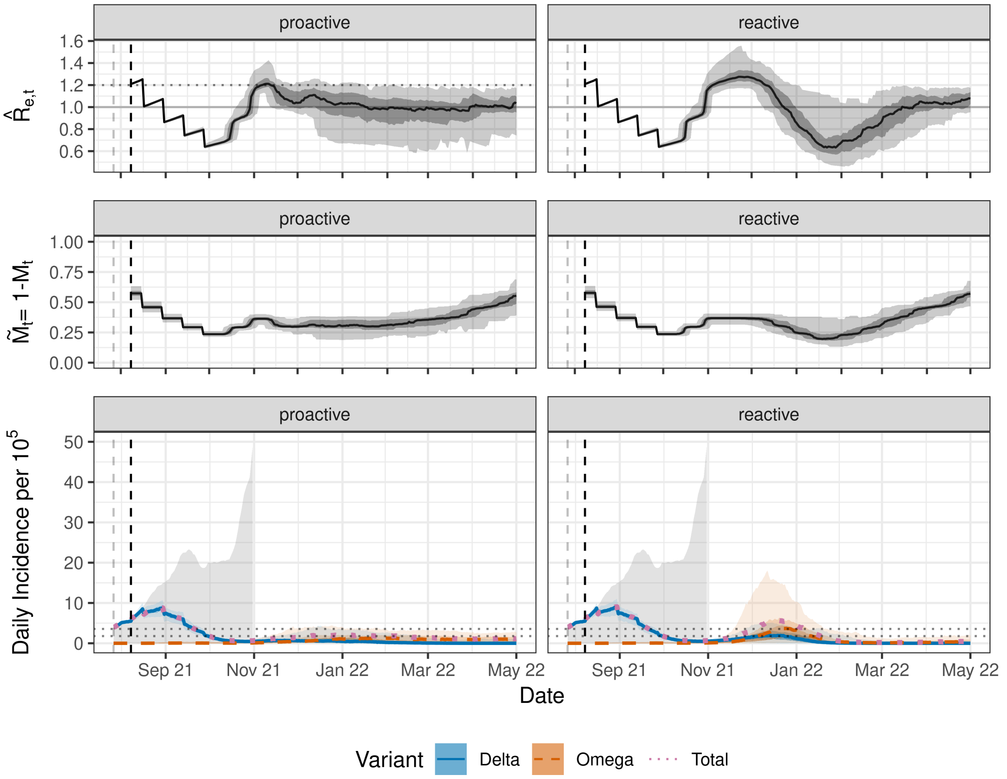

Fig D. Boosters primarily prevent a winter outbreak due to waning. With vaccine boosting, ρ_t^O declines fast enough so that large winter outbreaks are prevented – both under reactive and proactive control. Case thresholds are 25-50/100,000/14 days.

- We use reported incidence provided by the WHO dashboard [4].

  We assume all infections that happened in 2020 were with the wild type and we allocate the incidence of 2021 according to data provided by AGES [5] (see also Fig 15). Furthermore we scale this reported incidence to account for undetected infections based on values found in [6]. that actually get reported) of 0.42 which lies within the range of values investigated for Austria in [6].

- We use an estimate of the observed reproduction number using the R package EpiNow developed by [7]. These estimates are provided *via* [8].

The sampling procedure is as follows:

(1) Import data and choose the parameters as above.

(2) Simulate the overlap between vaccinated and previously infected individuals to determine the group sizes S_t^h.

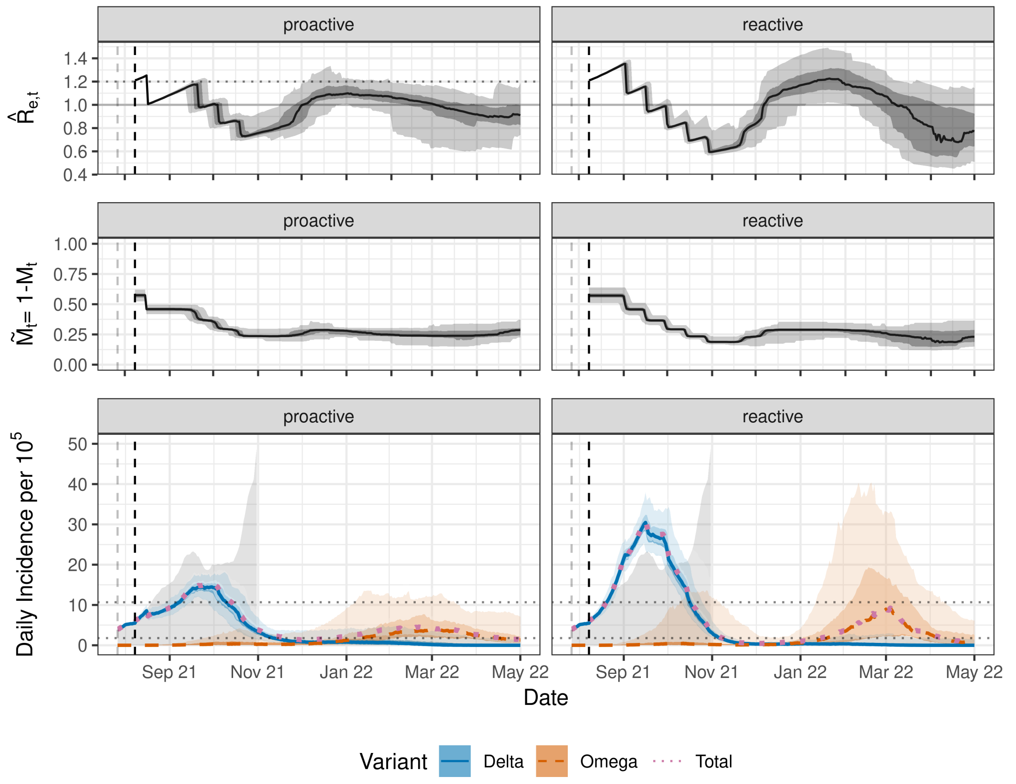

Fig E. Proactive control effectively prevents an Omega outbreak even with waning and high case thresholds. Case thresholds are 25-150/100,000/14 days, other parameters as is Fig 13.

(3) Choose initial incidence values, separated into the different variants (e.g. taking constant percentages or fitting a simple model like an exponential growth model, see Section Estimating Initial Variant Prevalence) as well as imported cases.

(4) Let t_0 the the the last day of observed data and let $\mathcal{R}_e$ denote the effective reproduction number observed on this day. For initializing the model we compute the initial mitigation $\tilde{M}_{t_0}$ using Equation 14 in the following way

$$\tilde{M}_{t_0} = \mathcal{R}_e W N \left(L_{t_0} \sum_{\mathcal{V}\in\mathscr{V}} \left(\sum_{g\in\mathscr{H}} \tilde{\gamma}^{g\mathcal{V}} |S_t^g| \sum_{h\in\mathscr{H}^{\mathcal{V}}} \sum_{m=1}^{\nu} \lambda^{\mathcal{V}} \mathcal{R}_0 I_{t-m}^h w_m \right)\right)^{-1} . \quad \text{(S1)}$$

(5) Sample the model as described in the Methods.

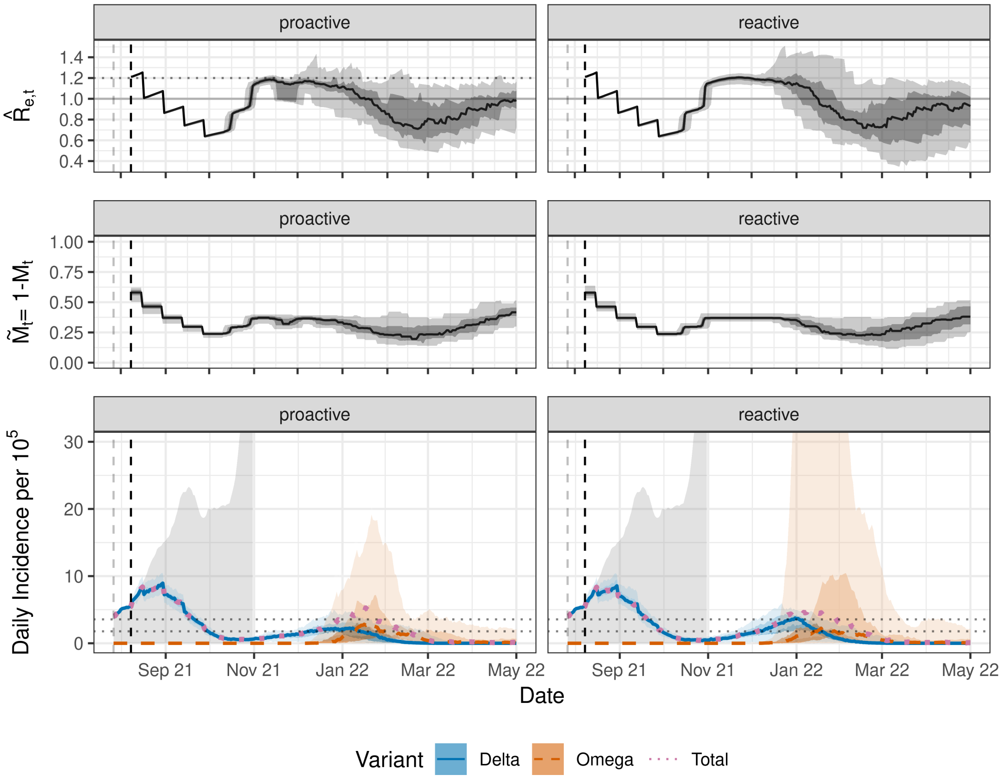

Fig F. Proactive control suppresses efficiently even a variant with a large $\rho_{\mathbf{t}}^{\mathcal{V}}$. In contrast, very large outbreaks are possible under reactive control. We modelled a modified Omega, which in addition to high immune escape (see Table 2), also has high basic reproduction number (same as Delta), while the mean of its generation interval is smaller at 3 days (CI: 1.5, 4.5). Case thresholds are 25-50/100,000/14 days. Both immunity waning and vaccine boosting are included. In contrast to all other simulations, here the import of Omega at the rate of 1/day starts later on 1.12.2021.

S3 Estimating Initial Variant Prevalence

In order to initialize the model with different variant prevalences as in the Delta simulations in Simulation and control of Delta, we use the following tentative estimation given the variant specific effective reproduction number $\mathcal{R}_{e,t}^{\mathcal{V}}$. For simplicity, we assume that initially all infections occur in non-interaction groups. and the only group infected with variant $\mathcal{V}$ will be group $g = 0$. Let us consider a simple exponential growth model

$$I_t^{\mathcal{V}} = c\left(\beta^{\mathcal{V}}\right)^t \tag{S1}$$

for a growth rate $\beta^{\mathcal{V}} > 0$. We equate this to the expected incidence given in (3), that is

$$I_t^{\mathcal{V}} = \mathcal{R}_{e,t}^{\mathcal{V}} \sum_{m=1}^{\nu} I_{t-m}^{\mathcal{V}} w_m = \mathcal{R}_{e,t}^{\mathcal{V}} \sum_{m=1}^{\infty} I_{t-m}^{\mathcal{V}} w_m$$

(where for the last equation we set the necessary w_m to 0) to get the following relation

$$c\left(\beta^{\mathcal{V}}\right)^t = \mathcal{R}_{e,t}^{\mathcal{V}} \sum_{m=1}^{\infty} w_t c\left(\beta^{\mathcal{V}}\right)^{t-m} \Leftrightarrow \frac{1}{\mathcal{R}_{e,t}^{\mathcal{V}}} = \sum_{m=1}^{\infty} w_t \left(\beta^{\mathcal{V}}\right)^{-m} = G_\omega\left(\frac{1}{\beta^{\mathcal{V}}}\right),$$

where $G_w(x) = \sum_{t=1}^{\infty} w_t x^t$ denotes the *probability generating function* of w.

Thus we can recover the growth rate $\beta^{\mathcal{V}}$ from the group reproduction number $\mathcal{R}_{e,t}^{\mathcal{V}}$ in the following way

$$\beta^{\mathcal{V}} = \left(G_w^{-1}\left(\frac{1}{\mathcal{R}_{e,t}^{\mathcal{V}}}\right)\right)^{-1}. \quad \text{(S2)}$$

Taking day t as a reference point we furthermore estimate the total growth rate $\hat{\beta}$ of the total incidence I_t using the observed reproduction number $\mathcal{R}_e$, precisely $\hat{\beta} = \left(G_w^{-1}\left(\frac{1}{\mathcal{R}_e}\right)\right)^{-1}$. If $p^{\mathcal{V}} \geq 0$ denotes the prevalence of variant $\mathcal{V}$ on day t, that is $I_t^{\mathcal{V}} = p^{\mathcal{V}} I_t$, then the estimated incidence of the previous days can be computed via

$$I_{t-s}^{\mathcal{V}} = p^{\mathcal{V}} \left(\frac{\hat{\beta}}{\beta^{\mathcal{V}}}\right)^s I_{t-s}.$$

Since the group specific reproduction number $\mathcal{R}_{e,t}^{\mathcal{V}}$ is usually not known it also needs to be tenatively estimated. For this we start with assuming constant variant prevalence first. Let $p^{\mathcal{V}_i} \geq 0$, $i = 1, \ldots, n$ such that $\sum_{i=1}^{n} p^{\mathcal{V}_i} = 1$ denote the prevalence of variant $\mathcal{V}_i$ within the total incidence I_t. Precisely $I_{t-s}^{\mathcal{V}_i} = p^{\mathcal{V}_i} I_{t-s}$ and $I_{t-s} = \sum_{i=1}^{n} p^{\mathcal{V}_i} I_{t-s}$ for $s = 0, \ldots, \nu - 1$. Using this constant initial incidence for each variant and using the observed reproduction number $\mathcal{R}_e$ we compute a tentative initial mitigation $\hat{M}_t$ using equation (S1). The variant specific reproduction number $\mathcal{R}_{e,t}^{\mathcal{V}}$ can then be estimated in the following way

$$\hat{\mathcal{R}}_{e,t}^{\mathcal{V}} = \hat{M}_t L_t N^{-1} \sum_{\substack{h \in \mathscr{C} \\ s.t.\, h = \overline{h}V}} \tilde{\gamma}^{gh} |S_t^g| \lambda^{\mathcal{V}} \mathcal{R}_0 \quad \text{(S3)}$$

and furthermore be used to compute the variant specific growth rate $\beta^{\mathcal{V}}$ using (S2).

S4 Estimating Daily Delta prevalence in Austria

For Austria the VOC prevalence data is provided only with weekly granularity [5]. In order to translate this data to daily granularity when initializing the Delta simulations in Simulation and control of Delta, we again use the simple exponential growth model (S1) and fit it to the weekly data. We take a reference time point $\bar{t}$ and use the following model

$$I_t^{\mathcal{V}} = \left(\beta^{\mathcal{V}}\right)^{t-\bar{t}} I_{\bar{t}}^{\mathcal{V}},\ t \in \mathbb{N},\ \beta^{\mathcal{V}} > 0.$$

If t is the end of a week, then the sum of all cases in this week is given via

$$\sum_{s=0}^{6} I_{t-s}^{\mathcal{V}} = \sum_{s=0}^{6} \left(\beta^{\mathcal{V}}\right)^{t-s-\bar{t}} I_{\bar{t}}^{\mathcal{V}} = \left(\beta^{\mathcal{V}}\right)^{t-\bar{t}} I_{\bar{t}}^{\mathcal{V}} \sum_{s=0}^{6} \left(\beta^{\mathcal{V}}\right)^{-s} = \left(\beta^{\mathcal{V}}\right)^{t-\bar{t}} I_{\bar{t}}^{\mathcal{V}} \frac{1-\frac{1}{(\beta^{\mathcal{V}})^7}}{1-\frac{1}{\beta^{\mathcal{V}}}}.$$

Let $\bar{J}_t^{\mathcal{V}}$ denote the cumulative weekly cases of variant $\mathcal{V}$ within the week that ends on day t. We then fit an exponential growth model via the following least squares method.

$$\inf_{(\beta^{\mathcal{V}}, I_{\bar{t}}^{\mathcal{V}}) \in \mathbb{R}^+ \times \mathbb{R}^+} \sum_t \left| \left(\beta^{\mathcal{V}}\right)^{t-\bar{t}} I_{\bar{t}}^{\mathcal{V}} \frac{1-\frac{1}{(\beta^{\mathcal{V}})^7}}{1-\frac{1}{\beta^{\mathcal{V}}}} - \bar{J}_t^{\mathcal{V}} \right|^2,$$

where we sum only over t corresponding to last days of the week.

To estimate the daily prevalence in Austria we fit this model to the weekly cumulative Alpha and Delta cases separately (assuming the prevalence of other variants to be negligible), thus daily Delta prevalence p_t^D can be recovered via

$$p_t^D = \frac{I_t^D}{I_t^A + I_t^D}.$$

We use the data provided by [5] between May 31, 2021 and July 21, 2021 to find that 20% Delta prevalence was most likely given on June 16, 2021 as seen in Fig G.

References

1. Richter L, Schmid D, Chakeri A, Maritschnik S, Pfeiffer S, Stadlober E. Schätzung des seriellen Intervalles von COVID19, Österreich; 2020.

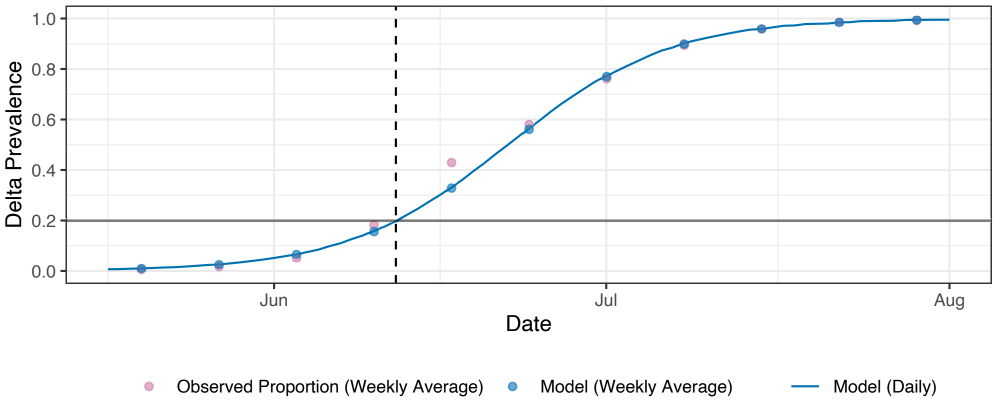

Fig G. Reported and Estimated Delta prevalence in Austria between May and August 2021.

2. Statistik Austria. Population of Austria at the beginning of the year 2021 (regional status of 2021); 2021. Available from: `https://data.statistik.gv.at/data/OGD_bevstandjbab2002_BevStand_2021.csv`.

3. Bundesministerium für Soziales, Gesundheit, Pflege und Konsumentenschutz. COVID-19 vaccination data for Austria; 2021. Available from: `https://info.gesundheitsministerium.gv.at/data/COVID19_vaccination_doses_timeline.csv`.

4. World Health Organization. WHO COVID-19 Dashboard; 2021. `https://covid19.who.int/WHO-COVID-19-global-data.csv`.

5. Agentur für Gesundheit und Ernährungssicherheit GmbH. SARS-CoV-2-Varianten in Österreich; 2021. `https://www.ages.at/themen/krankheitserreger/coronavirus/sars-cov-2-varianten-in-oesterreich/`.

6. Rippinger C, Bicher M, Urach C, Brunmeir D, Weibrecht N, Zauner G, et al. Evaluation of undetected cases during the COVID-19 epidemic in Austria. BMC Infectious Diseases. 2021;doi:10.1186/s12879-020-05737-6.

7. Abbott S, Hellewell J, Thompson RN, Sherratt K, Gibbs HP, Bosse NI, et al. Estimating the time-varying reproduction number of SARS-CoV-2 using national and subnational case counts. Wellcome Open Research. 2020;5(112):112.

8. Eder M, Hermisson J, Hledik M, Hütter C, Iofinova E, Pisupati R, et al. EpiMath: R-Nowcasting for Austria; 2021. Available from: https://epimath.at/generated/bigr_estimates.rds.
