# Supplementary material for: Robust models of disease heterogeneity and control, with application to the SARS-CoV-2 epidemic
Source: PLOS Glob Public Health. 2022 May 9;2(5):e0000412. doi: 10.1371/journal.pgph.0000412 (PMC10021456; doi:10.1371/journal.pgph.0000412)
Supplement: Attachment — Submitted filename: Response to Reviewers.pdf [file pgph.0000412.s002.pdf]

Response to Reviewers

We thank the reviewers for their detailed and very helpful comments. Through addressing their concerns, we believe the paper has been both strengthened and clarified. Below we describe the changes made in response to the points that were raised.

Before addressing each comment in turn, we want to highlight the largest changes in this version. We have added both a methods and results subsection that addresses immunity waning and booster shots. The goal here is twofold: firstly, to incorporate a feature of growing importance to COVID-19 modeling in order to strengthen our results; and secondly, to demonstrate increased usefulness of the $\rho^{\mathcal{V}}$ parameter. Furthermore, we switched graphics from less strict case thresholds of 25-150/100,000/14 days to 25-50/100,000/14 days. This is done to strengthen the claim that merely using stricter thresholds of the wrong statistic does not efficiently control outbreaks.

Reviewer # 1

- *the authors should describe how $\hat{R}_{e,t}$ is derived in practice.* This is now addressed at the end of the section on initialization and can be found on page 19. After this point, all components of the model have been concretely specified.
- *constraints on ascertainment of infections.*

  In the original submission, infections were observed after a number of days equal to the "delay" parameter, which is set at either 7 or 21 days. In the revised version, we have also added a detection ratio of $1/1.4 = .714$ to separate infections and observed cases.
- *words "cases" and "infections" are used interchangeably.*

  We have now addressed this: throughout, 'cases' denote the reported cases, which the controllers act on, while 'infections' propagate in the model.
- *The results in the Abstract and Discussion is that the proactive controller is more effective. This is an obvious result. Instead, % reduction in cumulative infections, % reduction in time under different stringencies of NPIs should be reported in the Abstract and results*

  We have added a more rigorous, quantitative comparison of the performance of controls, comparing both total infections and peak cases under quarantine (isolation). Furthermore, new graphs have been added to clearly demonstrate the differences in the distribution of NPI strength used by reactive and proactive controls. These demonstrate that the proactive

control is able to find a "balance point" between suppressing an outbreak and over-using NPIs. This holds true regardless of whether it observed information with a greater delay. In addition, the proactive controller with high case thresholds still performs better than the reactive controller with low case thresholds. These provide additional conceptual results, even before reporting the quantitative measures. One large take-away is that even when median values are similar, quantiles of predicted infections or quarantined cases differ greatly: reactive control fails to provide reliable control, as simulation trajectories can still experience large outbreaks. We have added several quantitative estimates with corresponding confidence intervals throughout the paper. Due to word-count constraints as well as out of concern that such quantitative comparisons could be taken out of context, these estimates were not put in the abstract. In particular, the estimates depend on the length of the simulation, the number of outbreaks experienced, and even simulation start time as we simulate over the winter where seasonality is strongest. Instead, we have strengthed our claims in the abstract. It now reads "We demonstrate that a controller that responds to the effective reproduction number in addition to case numbers is more efficient and effective in controlling new waves than monitoring case numbers alone. This not only reduces the median total infections and peak quarantine cases, but also controls outbreaks much more reliably: the observations cluster much more strongly around the median. Such a controller entirely prevents rare but large outbreaks."

- All other minor comments have been fixed in the manuscript.

Reviewer # 2

- *the case positivity rate and $\hat{R}_{e,t}$ computed by the model is the true underlying value assuming 100% detection rate.*

  The statistics observed by controllers are now determined by a detection ratio that is assumed fixed throughout the simulation (although we initialize the simulation with historical periods with different detection ratios). We do not implement a model for estimating a time-varying detection ratio as the simulation progresses; this is also now addressed in the discussion. Furthermore, cases are observed 7 or 21 days after infection to account for the delay between infection and observation.

- *There are more meaningful hypothetical questions that can be queried by the model. For instance, what is the extent and duration that NPIs are needed to control future waves for countries with different vaccination coverage? Furthermore, what differences in future outbreaks would we expect for countries such as Singapore and New Zealand, which have previously kept local transmissions low (minimal infection-induced immunity) and had/expected to have high vaccination coverage, compared to others such as the U.K. that had experienced higher infection numbers.*

  In response to the query, we have substantially expanded the subsection on Comparing Variants (pages 23-25). Notably, we included a graph to demonstrate different progression of $\rho^{\mathcal{V}}(r)$ depending on the different types

of immune landscapes that could be observed for countries with different infection and vaccination histories. These demonstrate not only different rankings of variants of concern, but also significantly different reproduction numbers $\rho^{\mathcal{V}}$. $\rho^{\mathcal{V}}$ is the conceptual driver of outbreaks in our model as it summarizes the immune landscape. Furthermore, herd immunity is understood in context of current mitigation, meaning that $\rho^{\mathcal{V}}$ can be used to guide NPIs use in order to suppress an outbreak. Populations with higher $\rho^{\mathcal{V}}$ will need either need higher NPIs in order to suppress an outbreak, or require very strict border controls to prevent importing and allowing community spread of a high-risk variant.
