# Supplementary material for: Robust models of disease heterogeneity and control, with application to the SARS-CoV-2 epidemic
Source: PLOS Glob Public Health. 2022 May 9;2(5):e0000412. doi: 10.1371/journal.pgph.0000412 (PMC10021456; doi:10.1371/journal.pgph.0000412)
Supplement: Attachment — Submitted filename: responseToReview.pdf [file pgph.0000412.s003.pdf]

Response to Reviewers

We would like to thank the reviewers and the editor for their clarifications and suggestions to improve the manuscript. The comments are addressed in turn below.

Following the suggestion of the editor, we made the text accessible to an 'average global public health' reader. We have added a "Summary of Methods" section after the introduction and presented the detailed Methods sections at the end of the paper. We believe that the methods section cannot be separated entirely from the main text for two primary reasons. First, there are many details which cannot be captured in a summary because it requires too much notation to be satisfactorily introduced. Furthermore, the methods section itself contains significant justification and discussion for many modeling decisions that were made. Second, contrary to the paper the editor suggested, the methods constitute a significant intellectual contribution. The paper cited was able to push details of regression and Gaussian process models to the appendix, as these were not formally developed in the paper. Instead, they were used and applied in novel ways to new data. On the other hand, our modeling framework and simulation is itself the contribution, and relegating it to the supplemental material sidelines its importance. Lastly, as reviewers have lauded its clarity, we believe that it will still be of benefit to those interested in learning about the method. We trust this arrangement is beneficial to the readers, and in line with the standards of PLoS articles (the last author's paper in PLoS Biology follows this arrangement, too: https://doi.org/10.1371/journal.pbio.2005372).

Reviewer # 1

- *Clarification on how $\hat{R}_{e,t}$ is derived in practice with many unknown parameters.* We addressed this concern in two places. First, at the end of the initialization section (last paragraph, p. 44) we discuss the importance of sampling parameters from a suitable range of values which capture the uncertainty. In fact, this is what we do in our simulations as well as parameters are not known with certainty. This is also used again in supplemental material to modify the generation time to explore the effect of this uncertainty. Second, the discussion also mentions the ability to estimate the reproduction number of hospitalizations. We show that using such delayed information does not significantly compromise the ability of the proactive controller. As hospitalizations are known much more concretely, there are fewer unknowns to cause problems. Specifically, we state (top paragraph, p. 27): "If the ascertainment bias for detecting cases is changing, one can use $R_{e,t}$ estimated from the hospitalization rate after taking age structure into account. While inducing NPIs when hospital capacity is reached is a

severely delayed reactive controller and performs poorly, reacting to $R_{e,t}$ estimated using such delayed information still provides reasonably efficient control - as shown in section Controller Types."

- *systematize the quantitative results comparison in a table.*

  The end of the results section now includes a table summarizing the quantitative results presented in the paper, which also points out to the respective Figures. In addition, we grouped together the density figures which illustrate that the mitigation using proactive (vs. reactive) control is always more "balanced", creating panel Fig 8.

- *drop the use of "Omega" to refer to the Omicron variant.*

  We added more text to the manuscript to explain our decision to explicitly distance our results from Omicron (p. 6,7,20). While we have attempted to make the resistance parameters as accurate to Omicron as possible given the information available during the review process, there are other potential differences which are not fully explored or only touched upon in the supplemental. For example, our discussion of hospitalizations is complicated by the fact that Omicron leads to differential hospitalization rates. In the end, we take the cautious approach of focusing on the general conclusions and use of the model and distancing ourselves from being proverbially harpooned by media sources should we fail to mimic Omicron entirely.

Reviewer # 2

We are glad that Reviewer # 2 is happy with our revised MS, and would like to thank her/him for the encouraging remark.
